# Deep Learning Classification of Uveal Melanoma and Metastatic Carcinoma on Cytology Specimens: A Single-Center Experience

**DOI:** 10.1167/iovs.67.11.47

**Published:** 2026-09-24

**Authors:** Max Jackson, Helen Kalirai, Rumana Hussain, Heinrich Heimann, Yalin Zheng, Sarah E. Coupland

**Affiliations:** 1 Department of Eye and Vision Science, University of Liverpool, William Henry Duncan Building, Liverpool, Merseyside, United Kingdom; 2 Liverpool Ocular Oncology Center, University Hospitals of Liverpool Group, Mount Vernon St, Liverpool, Merseyside, United Kingdom; 3 Liverpool Center for Cardiovascular Science, University of Liverpool, William Henry Duncan Building, Liverpool, Merseyside, United Kingdom

**Keywords:** choroidal melanoma, ocular metastasis, deep learning, cytospin

## Abstract

**Purpose:**

Differentiating between a choroidal melanoma and metastatic carcinoma to the choroid on cytological specimens presents significant diagnostic challenges due to subtle morphological differences in the sparse cells available in the sample and the increasing scarcity of specialist ocular pathologists. This study developed and evaluated deep learning models for automated classification of May-Grünwald-Giemsa–stained cytospin whole slide images (WSIs) to support diagnostic decision-making.

**Methods:**

Cytospin WSIs from 87 patients (50 with choroidal melanoma and 37 with metastatic carcinoma to the choroid) who underwent trans-scleral fine-needle aspiration biopsy at the Liverpool Ocular Oncology Center were digitized and tiled into 256 × 256 pixel patches. Variants of U-Net architectures were trained on 692 manually annotated tiles for tumor cell segmentation. The diagnostic decision model was developed and evaluated using different classification frameworks for best diagnostic performance: five convolutional neural network, three vision transformer (ViT) models, and seven multiple instance learning (MIL) architectures, with and without three preprocessing approaches (original, segmented, and mask-out tiles). Performance was assessed using three-fold patient-level cross-validation, with 20% of the cohort (*n* = 17 of the 87 patients) held out as a final independent test set.

**Results:**

The U-Net achieved strong segmentation performance with a mean absolute error of 1.91 ± 1.31 µm, precision of 0.80 ± 0.17, and recall of 0.76 ± 0.20. Double-tier feature distillation–MIL with mask-out tiles and a fine-tuned Swin-Tiny/4 back-bone showed the highest patient-level area under the curve (AUC) of all models (AUC, 0.96; 95% confidence interval, 0.83–1.00), while DenseNet121 had the highest patient-level accuracy (0.84). Convolutional neural network models performed more consistently than other frameworks over all preprocessing methods, while MIL architectures with domain-specific features achieved the strongest overall AUC on mask-out tiles. ViTs varied somewhat over the different preprocessing techniques with mask-out tiles achieving the highest ViT performance (Swin-Tiny/4; AUC, 0.87).

**Conclusions:**

This single-center experience demonstrates the application of deep learning to cytospin WSIs in ocular oncology, establishing a complete pipeline from tumor cell segmentation to slide-level classification. The framework provides a foundation for artificial intelligence–assisted cytopathology that could accelerate diagnosis, particularly in centers lacking specialist ocular pathology expertise, and may be adaptable to other rare cytological specimens.

Uveal melanoma is a rare but aggressive cancer affecting approximately 6 people per million per year.^1^ Ninety percent of all cases arise in the choroid,^2^ with metastasis occurring in approximately 50% of all cases over their lifetime.^3^ Post metastatic progression, the median survival time is less than 6 months.^4^ Metastatic carcinomas (mCAs) can spread to the choroid, most commonly arising from primary breast or lung cancers.^5^ Ocular mCAs are also associated with a poor patient prognosis, with a median survival time of less than 6 months from diagnosis of the disseminated disease to death.^5^ Choroidal melanoma (CM) and mCA are diagnosed primarily by ocular oncologists using a variety of nonionizing imaging techniques. Each imaging method provides differing information about the lesion(s) that can be integrated for a clinical diagnosis. In some cases, these images are still not sufficient for a definitive diagnosis. At selected specialist ocular oncology centers, such as the Liverpool Ocular Oncology Center, intraocular biopsies are routinely undertaken to determine the nature of such lesions for either diagnostic or prognostic purposes. Cytology preparations are stained with May–Gru¨newald–Giemsa (MGG) to highlight tumor cells and enable the pathologist to examine their morphological features. Melanoma cells typically demonstrate dark mauve-colored ovoid nuclei with discrete nucleoli and a less intense narrow cytoplasmic rim that may contain melanin pigment. Red blood cells also appear with a lighter stain and can be sometimes seen to encircle the tumor cells in a rosette-like pattern. Occasional lymphocytes and macrophages may be seen in the background. In contrast, mCA cells have a dark, typically larger pleomorphic nucleus with a variably sized pale cytoplasmic rim. No pigment is present in the cytoplasm of the mCA cells, but they may contain mucin, if from a primary adenocarcinoma. Immunostaining may be required to help differentiate a CM from an mCA. However, intraocular biopsies may yield limited material, not allowing for additional testing. This highlights the need for a tool that can aid specialist pathologists to ensure accurate diagnostic decisions. The widespread adoption of digital pathology has fundamentally transformed histopathological practice, with an increasing number of centers transitioning to whole slide imaging (WSI) for diagnostic purposes. This digital transformation has created extensive repositories of pathological images, providing unprecedented opportunities for developing computational diagnostic tools through artificial intelligence (AI). AI and deep learning (DL) have demonstrated significant promise across multiple domains of computational pathology, with recent advances showing great potential for augmenting diagnostic accuracy and efficiency. However, applications have focused predominantly on histopathological WSIs, with comparatively limited development of AI tools for cytological specimens. This disparity likely reflects the unique challenges posed by cytology, including smaller available training datasets with varying cellular density on slides and the inherent difficulty of both cell segmentation and classification tasks in sparse cellular environments. While DL models have been applied to cytological specimens in other domains, such as bone marrow morphology, no studies have addressed ocular cytospin WSIs. These specimens present distinct challenges, including extremely low cellularity, very small dataset sizes due to the rarity of intraocular biopsies, and a narrow but morphologically subtle differential diagnosis. This study evaluated the applicability of DL models to CM and mCA cytospin images, assessing their ability to accurately segment individual tumor cells and subsequently classify entire slides for differential diagnosis. Beyond its immediate clinical utility in supporting diagnostic decision-making, this work establishes a framework for applying DL to cytological specimens in ocular pathology and identifies optimal model architectures for this challenging domain.

## Methods

This study conformed to the principles of the Declaration of Helsinki and all procedures and methods relating to the data use were approved by the Health Research Authority under the REC Ref 24-SW-0150.

### Patients

A total of 87 patients who underwent trans-scleral fine-needle aspiration biopsy at the Liverpool Ocular Oncology Center between 2011 and 2025 were included in this study. Samples were obtained using with a 25G needle attached to a 20-mL syringe. The whole sample was transported in a CytoLyt Solution to the laboratory where using standard protocols a cytospin was created. These were stained with an MGG protocol previously optimized for ocular samples.^6^ Following specialist pathological review of MGG-stained cytospins, 50 patients were diagnosed with CM and 37 patients with mCA. Melanocytomas were excluded from this study. Of the 50 CM cases, 24 contained epithelioid cells in the specimen and 26 did not. Each patient sample contributed one WSI to the dataset with all cases selected to ensure that only pretreatment samples were used. The complete dataset composition through all stages of the preprocessing pipeline is presented in Table 1.

**Table 1.** Dataset Composition and Pre and Post Processing

Stage	CM	mCA	Total
Patients (WSI)	50 (60)	37 (40)	87
Tiles (*n ≥* 5)	148,194 (64)	83,483 (36)	231,677

Values are number (%). Tiles extracted at 256 × 256 pixels from WSI at ×20 magnification after removing tiles with >95% white space (background). *n* represents the number of segmented tumor cells per tile; only tiles with ≥5 cells were retained following U-Net application.

### Carcinoma Subtypes

Among the 37 mCA patients, the primary tumor site was classified as definite where histological or clinical confirmation was available, or probable where immunocytochemistry or clinical history suggested a likely origin but definitive confirmation was not possible (Table 2). The most common primary sites were the lung (*n* = 12) and the breast (*n* = 8). In eight cases, the primary site remained unknown at the time of ocular biopsy. The remaining cases arose from a range of primary sites, including the esophagus, prostate, thyroid, ovary, and upper gastrointestinal tract. This heterogeneity reflects the real-world clinical distribution of choroidal metastases and introduces morphological diversity into the mCA class, as carcinomas from different primary sites can exhibit varying cytological features.

**Table 2.** Primary Site of mCA Cases

Primary Site	Definite	Probable
Lung	8	4
Breast	8	0
Prostate	2	0
Esophageal	1	1
Gastroesophageal	0	1
Thyroid	1	0
Lung/thyroid	0	1
Lung/upper gastrointestinal tract	0	1
Ovarian	1	0
Unknown	8	0
Total	29	8

Cases were classified as definite where the primary site was confirmed, or probable where immunocytochemistry or clinical history suggested a likely origin.

### Digitization of Slides

WSIs were then taken using a ROCHE DP200 scanner at ×40 magnification and are stored as pyramidal .tif compressed files with JPEG compression at 95% quality. WSIs and clinical and genetic information were accessed through the Liverpool Ocular Oncology Bio-resource - Eye Cancer Artificial Intelligence Digital platform, which stores all data in a secure trusted research environment.

### Preprocessing

Due to their size, WSIs were first tiled into 256 *×* 256-pixel patches using the *×*20 magnification and stored as .png files. During this process, tiles with greater than 95% white space (background) were immediately removed to reduce the number of blank tiles and any tile with insufficient material. This initial triaging reduced the number of tiles to be processed from 1,718,777 to 742,023. To achieve this, tiles were converted to grayscale and the proportion of pixels with an intensity of greater than 220 was calculated. If this proportion exceeded 95%, they were removed. Additionally, tiles with low pixel variance (<150) and more than 40% white pixels were also rejected to filter out near-uniform regions. From the extracted tiles, a subset of 1000 tiles was randomly selected for manual annotation, comprising 500 tiles from each diagnostic category (CM and mCA) and equal number from each patient. Annotations were performed by marking the center point of each tumor cell with tiles containing fewer than five tumor cells excluded from the annotated dataset. Following this filtering process, 692 tiles remained (maintaining this equal split between the diagnoses) with complete centroid annotations of all visible tumor cells. The annotated dataset was then split into 80% training and 20% test sets before applying cell segmentation.

### Cell Segmentation Model

For cell segmentation, a U-Net architecture^7^ was implemented to identify individual tumor cells within cytospin tiles. The U-Net uses an encoder–decoder structure with skip connections to preserve spatial information during segmentation. Training masks were generated from the 692 manual cell centroid annotations by creating circular regions (radius of 10 pixels) around each marked cell. The model was trained for 100 epochs (batch size of 32) using combined binary cross-entropy and Dice loss, with data augmentation including rotations, flips, and color jittering. Tiles were kept at 256 *×* 256 pixels. Cell centroids were extracted from the predicted masks using local maxima detection (minimum 8-pixel separation). The trained U-Net was then applied to the remaining tiles, enforcing the minimum of five tumor cells per tile. In total, 231,677 segmented tiles, made up of 148,194 CMs and 83,483 mCAs, were carried forward for the classification task. All U-Net actions were performed on an NVIDIA GTX 4090 GPU.

### Segmentation Evaluation

The trained U-Net was evaluated on 20% of the 692 manually annotated tiles using the mean absolute error (MAE), which calculates the average Euclidean distance in pixels between each true centroid and its nearest predicted centroid:

(1) $$\begin{array}{c} \mathrm{MAE}=\frac{1}{n}\sum_{i=1}^{n}\min_{j}\sqrt{{\left(x_{i}-{\hat{x}}_{j}\right)}^{2}+{\left(y_{i}-{\hat{y}}_{1}\right)}^{2}}, \end{array}$$

where *n* is the number of true cell centroids, (*x_i_, y_i_*) represents the *i*-th true centroid coordinates, and ($\hat{x}$*_j_, ŷ_j_*) represents the *j*-th predicted centroid coordinates. Precision and recall were computed using a one-to-one tolerance-based matching approach with a 10-pixel threshold. All Euclidean distances are reported in micrometers using the scanner pixel size of 0.25 µm/pixel at *×*20 magnification (ROCHE DP200), so that 10 pixels corresponds with 2.5 µm. Ground truth and predicted centroids were paired using greedy nearest-neighbor assignment, ensuring that each centroid could only be matched once. A prediction *ĉ_j_* is matched to ground truth *c_i_* if they are the nearest unmatched pair with *d*(*c_i_, ĉ_j_*) *≤ t*, where *d*(*·, ·*) is the Euclidean distance and *t* = 10 pixels is the matching tolerance threshold. True positives are matched predictions, false positives are unmatched predictions, and false negatives are unmatched ground truth centroids. Precision and recall are then computed as:

(2) $$\begin{array}{c} \mathrm{Precision}=\frac{\mathrm{TP}}{\mathrm{TP}+\mathrm{FP}},\;\mathrm{Recall}=\frac{\mathrm{TP}}{\mathrm{TP}+\mathrm{FN}}. \end{array}$$

### Tolerance Threshold Validation

To validate the 10-pixel matching tolerance used in segmentation evaluation, we analyzed nearest-neighbor distances across all 692 annotated tiles containing 4327 tumor cells. The distribution of cell spacing showed a median nearest-neighbor distance of 24.7 pixels, with 90% of cell pairs separated by more than 10 pixels. This analysis confirms that the 10-pixel tolerance appropriately captures true-positive matches while minimizing false-positive pairings between spatially distinct cells. At twice this threshold (20 pixels), only 10.3% of cells had neighbors within this ambiguity zone, further supporting the 10-pixel tolerance.

### Classification of CM and mCA

A three-fold cross-validation strategy was implemented to robustly evaluate model performance at a patient level. Initially, 20% of the 87 patients (*n* = 17) were randomly selected and used as a held-out test set, with the remaining 80% (*n* = 70) used for cross-validation. Importantly, all tiles from a given patient were assigned exclusively to a single subset, ensuring that no tiles from the same patient appeared in both the training and testing sets. This patient-level splitting prevents data leakage and inflated performance estimates that could arise. Within each fold, patients were split into 80% training and 20% validation sets, with three different train–validation combinations across the three folds. This patient-level splitting ensured that all tiles from a given patient remained within the same subset, preventing bias from tile-level splitting. To investigate the impact of segmentation on classification performance, three datasets were created with identical cross-validation splits.

1. Original tiles: Unprocessed 256 *×* 256-pixel tiles extracted from WSIs (Fig. 1a).
2. Segmented tiles: U-Net outputs with cells segmented and background removed (Fig. 1c).
3. Mask-out tiles: Segmentation masks expanded by 8 pixels (2.0 µm) to capture additional cellular and local contextual information beyond the predicted centroid regions (Fig. 1b).

**Figure 1.**
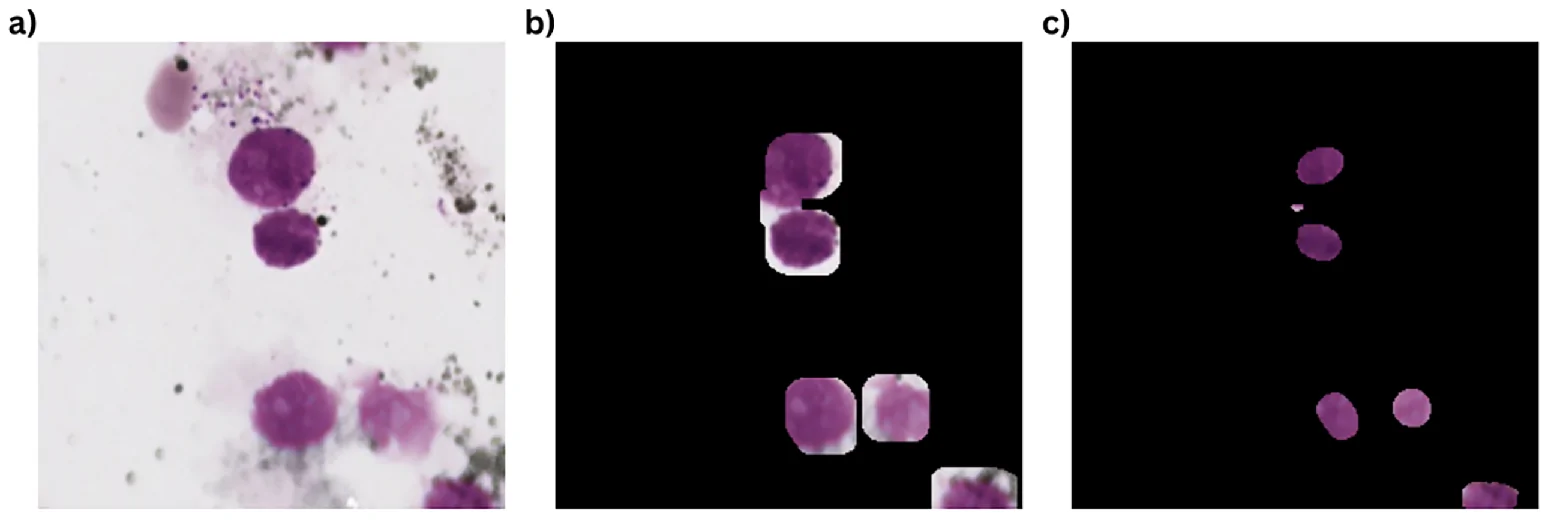
Comparison of tile preprocessing strategies for a 256 × 256-pixel tile from a CM patient stained with MGG. (**a**) Original unprocessed tile. (**b**) Mask-out tile with 8-pixel expansion applied to segmentation masks. (**c**) U-Net–segmented output with background removed.

The 8-pixel (2.0 µm) expansion in the mask-out approach was chosen based on the observed distribution of nearest-neighbor distances between annotated cell centroids. Analysis of the 4327 annotated cells across 692 tiles showed a median nearest-neighbor distance of 24.7 pixels (6.2 µm). The centroid detection mask has a radius of 10 pixels; with an 8-pixel expansion, the outer edge of each mask reaches 18 pixels from the centroid, leaving a median gap of approximately 6.7 pixels (1.7 µm) to the nearest adjacent cell. This expansion therefore maximizes the pericellular context retained while minimizing the overlap between the expanded masks of the neighboring cells. A larger expansion would risk merging masks from adjacent cells, introducing features from neighboring cells that could confound classification. Figure 1 highlights the differences between the same image in all three datasets with no segmentation, segmentation, and mask-out.

### Classification Models

A total of 15 models were evaluated from 3 different frameworks across 3 different preprocessing methods, all chosen for their distinct approaches to image analysis. Convolutional neural networks (CNNs) were selected as the standard DL approach, which are widely used in diagnostic pathology for their ability to recognize patterns and features in medical images. Vision transformers (ViTs) were included as a newer architecture that analyzes images by focusing on the relationships between different regions, which may help to identify sparse tumor cells distributed across cytospin slides. Multiple instance learning (MIL) models were chosen because they naturally suit pathology workflows where a diagnosis is made at the slide level based on features present anywhere within the specimen, without requiring the identification of every diagnostic cell. Following is a breakdown of each model architecture selected for this study.

### Training Protocol

All CNN and ViT models used transfer learning from ImageNet^8^ pretrained weights with final layers modified for binary classification. Images were resized to 224 *×* 224 pixels with standard normalization. Tiles were extracted at 256 *×* 256 pixels (64 µm *×* 64 µm at *×*20 magnification), so resizing to 224 *×* 224 represents a modest 12.5% reduction in spatial dimensions. This was preferred over center cropping, which would discard up to 25% of each tile and risk excluding cells near tile borders—a concern in the sparse preparations used here. The same resizing is applied consistently across all architectures, ensuring valid comparisons. Training used three-fold cross-validation with AdamW optimization (learning rate 1 *×* 10*^−^*^5^, batch size 128), cross-entropy loss, and early stopping (5-epoch patience, 30-epoch maximum) on an NVIDIA H100 GPU. Performance was assessed using the patient-level area under the curve (AUC) of the receiver operating characteristic curve and patient-level accuracy, sensitivity, and specificity. The MIL architectures used precomputed tile-level features from the fine-tuned Swin-Tiny/4 backbone, as detailed elsewhere in this article.

### CNNs

Five CNN architectures were evaluated for the binary classification of cytospin images into CM and mCA: EfficientNet-B0,^9^ ResNet50,^10^ DenseNet121,^11^ MobileNetV2,^12^ and VGG16.^13^ These models were selected for their proven performance in medical image analysis and varying computational requirements.

### ViTs

To assess attention-based architectures, three transformer models were implemented: ViT-Base/16,^14^ DeiT-Base/16,^15^ and Swin-Tiny/4.^16^ Model selection encompassed diverse transformer strategies, including standard patch tokenization, data-efficient training with distillation, and hierarchical shifted-window mechanisms.

### MIL Models

MIL is a weakly supervised learning paradigm particularly suited to WSI analysis, where slide-level diagnostic labels are available, but precise annotations of diagnostic features are absent. In the MIL framework, each WSI is treated as a bag containing multiple instances (image tiles), with the slide-level label assigned to the entire bag rather than individual tiles. This approach is well-aligned with histopathological diagnosis, where the overall slide classification may depend on the presence or absence of diagnostic features distributed across the specimen, even when their precise locations are unknown. All MIL architectures used an identical feature extraction step to ensure a fair comparison. Tile-level features were extracted using the fine-tuned Swin-Tiny/4 backbone (the same model evaluated in the ViT experiments, trained end-to-end on this dataset), with tiles resized to 224 *×* 224 pixels. Features were precomputed once per patient and stored as 768-dimensional cached embeddings; no tile cap was applied and all available tiles were used during training, with a minimum requirement of 5 tiles for inclusion. Seven MIL architectures were evaluated (Mean-pooling, Max-pooling, Attention,^17^ Gated attention,^17^ Transformer,^18^ dual-stream maximum self-attention MIL,^19^ and double-tier feature distillation [DTFD]),^20^ covering a wide range of aggregation strategies from simple pooling to complex attention-based and hierarchical approaches. All models were trained using the AdamW optimizer with early stopping based on a validation AUC. Class imbalance was addressed through inverse frequency weighting with the same cross-validation splits used for both the CNN and ViT architectures.

### Model Evaluation

Model performance was evaluated using a nested validation strategy to ensure robust assessment while preventing data leakage. During the three-fold cross-validation training phase, each model's performance was assessed on the held-out validation fold using the AUC, accuracy, sensitivity, and specificity. Following cross-validation, the fold achieving the highest validation AUC for each model architecture was selected, and that specific trained model was then evaluated on a separate held-out internal test set that had been withheld from all training and validation procedures. Cross-validation was used as a model selection mechanism to identify the best-performing checkpoint for each architecture; the held-out internal test set provides the unbiased performance estimate, as it was not used in any way during training or fold selection. A limitation of this approach is that variability in performance across folds is not reported; the selected fold may be optimistically biased relative to a fold-averaged estimate, although the held-out test set evaluation remains independent. Throughout this article, this subset is referred to as the “held-out internal test set” to distinguish it from an external (geographically or institutionally independent) validation cohort, which this study does not include. The held-out internal test set performance metrics represent the final reported results for each architecture. The patient AUC served as the primary metric for comparing model performance, because it provides a threshold-independent measure of classification ability. For the CNN and ViT models, the per-patient score used to compute the receiver operating characteristic curve was the mean tile-level predicted probability across all tiles belonging to that patient; the AUC was then calculated over these patient-level mean probabilities. For the MIL models, the patient-level score is the slide-level probability output directly by the MIL aggregator, which is inherently a bag-level (patient-level) classifier. Class activation maps^21^ were implemented for the conventional classification models to improve interoperability by highlighting the key regions used for classification. Patient-level accuracy was calculated for both the CNN and MIL architectures, with the CNN having a majority voting system (>50% of tiles were classified as one category) and the MILs each having their own method of bagging.

### Full Pipeline

Figure 2 provides a full breakdown of the segmentation and classification pipeline, with the left-hand side being the segmentation and right the classification.

**Figure 2.**
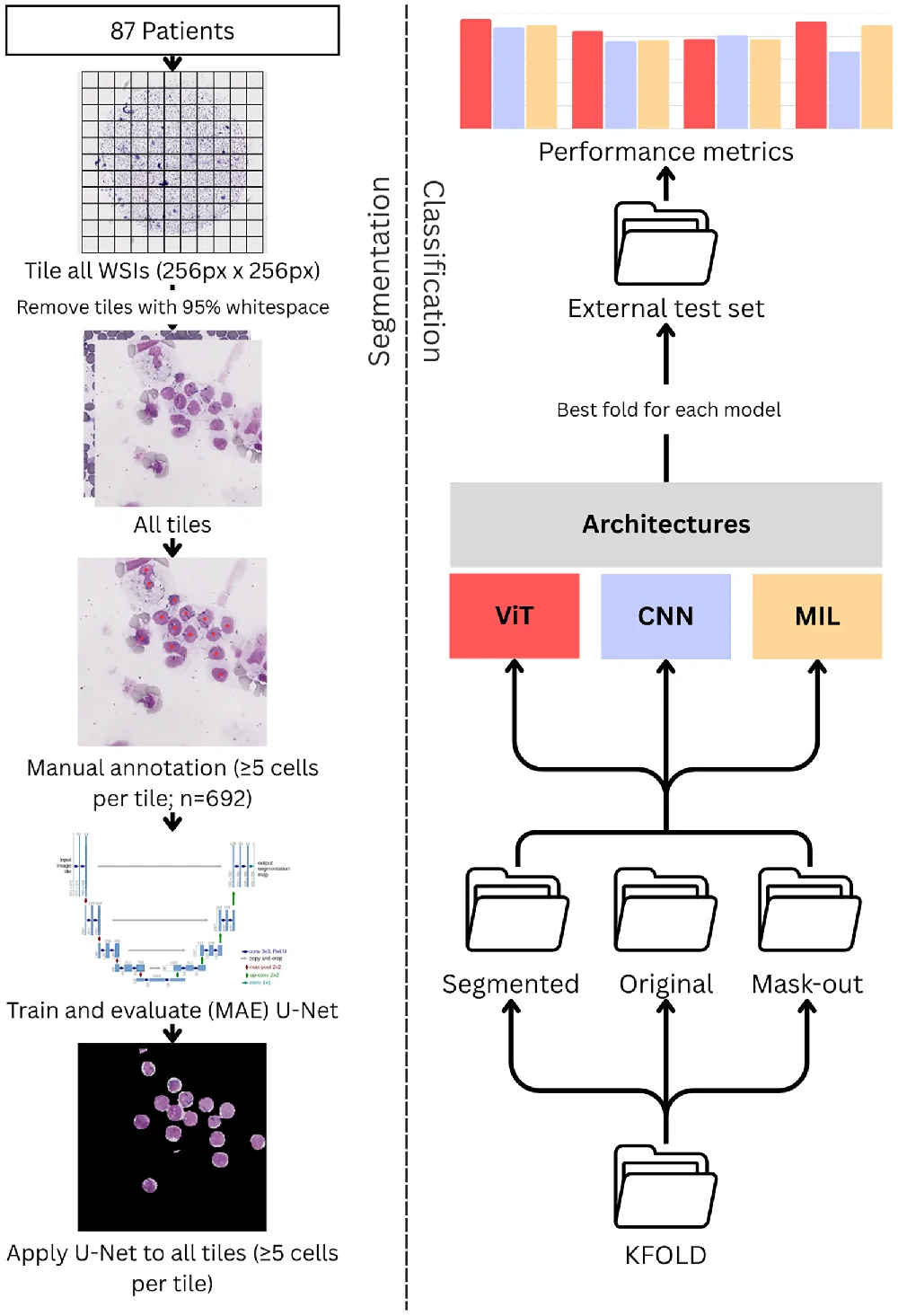
DL pipeline for cytospin WSI classification. WSIs from 87 patients are tiled, filtered, and processed through U-Net segmentation. Three input types (segmented, original, and mask-out) are classified using ViT, CNN, and MIL architectures with three-fold cross-validation. See Methods for detailed parameters and evaluation metrics.

## Results

### Segmentation

The U-Net cell segmentation model served as a preprocessing step to generate the three tile variants (original, segmented, and mask-out) used in the downstream classification experiments; the results presented elsewhere in this article validate this component of the pipeline. The U-Net demonstrated a strong performance for tumor cell centroid detection on 692 annotated tiles. On the validation set (20% hold-out), the model achieved a MAE of 1.91 *±* 1.31 µm, with a precision of 0.80 *±* 0.17 and recall of 0.76 *±* 0.20 using a 2.5-µm matching tolerance. Eighty percent of the predicted centroids corresponded with manually annotated cell positions within the tolerance threshold, while the model successfully detected 76% of all manually annotated cells. The training set performance showed higher precision (0.93 *±* 0.09) with similar recall (0.78 *±* 0.21), suggesting good generalization with minimal overfitting. The variability in performance metrics reflects the inherent detection difficulty across images, with some tiles containing overlapping cells, variable staining intensity, or cellular debris that increase localization uncertainty.

### Classification

Table 3 displays the comprehensive held-out internal test set performance across all evaluated architectures and preprocessing conditions. The mask-out tiles outperformed both nonsegmented and segmented approaches across the CNN and ViT frameworks based on the patient-level AUCs. The MILs showed a moderate performance on the original tiles and very poor performance on both the segmented and mask-out tiles in both patient-level AUC and accuracy. The ViT framework showed the best overall patient-level AUC and performed best on the mask-out tiles. Performance varied considerably between architectures within each framework, with some models showing greater sensitivity/specificity and other failing to classify. A detailed analysis of each framework is presented in the following sections.

**Table 3.** Held-Out Internal Test Set Performance Across All Model Frameworks and Preprocessing Conditions

Preprocessing	Model	AUC [95% CI]	Accuracy	Sensitivity	Specificity
CNN					
Original	DenseNet121	0.73 [0.44–0.97]	0.74	0.56	0.85
	EfficientNet-B0	0.71 [0.43–0.95]	0.74	0.65	0.80
	MobileNetV2	0.74 [0.48–0.95]	0.83	0.72	0.91
	ResNet50	0.73 [0.45–0.94]	0.78	**0.84** ^*^ ^,^ ^†^	0.74
	VGG16	0.79 [0.53–0.97]	0.83	**0.84** ^*^ ^,^ ^†^	0.81
Mask-out	DenseNet121	0.79 [0.50–0.98]	**0.84** ^*^ ^,^ ^†^	0.76	0.89
	EfficientNet-B0	0.77 [0.50–0.97]	0.77	0.56	0.90
	MobileNetV2	0.79 [0.53–0.98]	0.75	0.50	**0.91** ^†^
	ResNet50	0.79 [0.50–0.99]	0.76	0.55	0.90
	VGG16	**0.80** ^†^ [0.54–0.98]	0.77	0.63	0.87
Segmented	DenseNet121	0.71 [0.42–0.95]	0.64	0.47	0.75
	EfficientNet-B0	0.60 [0.30–0.87]	0.70	0.47	0.85
	MobileNetV2	0.64 [0.33–0.90]	0.71	0.46	0.88
	ResNet50	0.71 [0.43–0.95]	0.70	0.53	0.81
	VGG16	0.71 [0.45–0.94]	0.70	0.42	0.88
ViT					
Original	DeiT-Base/16	0.77 [0.49–1.00]	**0.80** ^†^	0.69	**0.87** ^†^
	Swin-Tiny/4	0.61 [0.30–0.88]	0.74	**0.79** ^†^	0.71
	ViT-Base/16	0.69 [0.41–0.93]	0.75	0.69	0.80
Mask-out	DeiT-Base/16	0.79 [0.52–0.98]	0.76	0.65	0.84
	Swin-Tiny/4	**0.87** ^†^ [0.64–1.00]	0.77	0.70	0.83
	ViT-Base/16	0.74 [0.46–0.95]	0.74	0.61	0.82
Segmented	DeiT-Base/16	0.63 [0.32–0.89]	0.71	0.49	0.86
	Swin-Tiny/4	0.76 [0.48–0.97]	0.71	0.47	0.86
	ViT-Base/16	0.53 [0.22–0.83]	0.70	0.46	0.85
MIL (Swin-Tiny/4 backbone)					
Original	Max	**0.81** ^†^ [0.57–1.00]	0.47	**1.00** ^†^	0.10
	DSMIL	0.46 [0.17–0.78]	0.41	0.71	0.20
	Attention	**0.81** ^†^ [0.54–1.00]	0.65	0.14	**1.00** ^†^
	Gated Attention	0.67 [0.36–0.92]	0.53	0.86	0.30
	Transformer	0.64 [0.36–0.89]	0.59	0.29	0.80
	Mean	0.63 [0.32–0.88]	0.59	0.43	0.70
	DTFD	0.73 [0.44–0.97]	**0.71** ^†^	0.71	0.70
Mask-out	Max	0.41 [0.12–0.72]	0.41	0.29	0.50
	DSMIL	0.91 [0.74–1.00]	0.76	0.57	0.90
	Attention	0.86 [0.62–1.00]	0.76	0.57	0.90
	Gated Attention	0.90 [0.70–1.00]	0.76	0.57	0.90
	Transformer	0.86 [0.63–1.00]	0.76	0.43	**1.00** ^†^
	Mean	0.87 [0.66–1.00]	**0.82** ^†^	**0.71** ^†^	0.90
	DTFD	**0.96** ^*^ ^,^ ^†^ [0.83–1.00]	0.71	0.29	**1.00** ^†^
Segmented	Max	0.67 [0.37–0.92]	0.59	0.43	0.70
	DSMIL	0.66 [0.38–0.90]	0.59	0.00	1.00
	Attention	0.77 [0.48–1.00]	0.59	0.00	1.00
	Gated Attention	0.73 [0.45–0.97]	0.59	0.00	1.00
	Transformer	0.56 [0.23–0.89]	0.47	**0.57** ^†^	0.40
	Mean	**0.80** ^†^ [0.52–1.00]	0.59	0.00	1.00
	DTFD	0.70 [0.40–1.00]	0.59	0.00	1.00

CI, confidence interval; DSMIL, dual-stream maximum self-attention MIL.

Patient-level metrics were determined by majority voting (CNN/ViT) or model-specific aggregation (MIL). MIL tile-level features were extracted using the fine-tuned Swin-Tiny/4 backbone. AUCs and 95% CIs were computed using 2000 patient-level bootstrap resamples.

* Values are the best overall performing model out of any architecture and preprocessing.

† Values are the best per framework.

### CNN Performance

Classification performance from all five CNN architectures is shown in Table 3, where VGG16 showed the best overall performance for patient AUC (0.80) and DenseNet121 had the best accuracy (0.84), both using the mask-out tiles. Both the original and mask-out tiles performed statistically significantly better (*P* *<* 0*.*05 and *P* *<* 0*.*001, respectively) than the segmented tiles, with no statistically significant differences between the original and mask-out tiles. Full segmentation hindered the classification; however, the removal of some of the noncellular features on the tiles did improve performance, as shown in the mask-out results.

### ViT Performance

ViT architectures (Table 3) demonstrated good overall performance on the mask-out tiles, with Swin-Tiny/4 achieving the highest patient-level AUC (0.87). DeiT-Base/16 achieved the highest accuracy (0.80) of all ViTs when applied to the original tiles. There were no statistically significant differences between the preprocessing methods and patient AUC, with the only statistically significant difference (*P* *<* 0*.*05) being between the original and segmented tiles in accuracy.

### MIL Performance

The MIL models (Table 3) performed worse than both the CNN and ViT architectures with mask-out and segmented tiles performing statistically significantly worse (*P* *<* 0*.*001) than the original tiles in both patient AUC and accuracy. The max pooling aggregation achieved the highest performance on original tiles (AUC, 0.81; accuracy, 0.47). Among the MIL approaches, the highest AUC on the original tiles was achieved jointly by Max and Attention aggregation (AUC, 0.81), while DTFD^20^ achieved the highest accuracy on original tiles (accuracy, 0.71). On segmented tiles, DTFD and several other aggregators achieved perfect specificity (1.00) with near-zero sensitivity (0.00), consistent with model collapse toward the majority class in the absence of discriminative features.

### Comparisons

Figure 3 shows the top 10 best-performing models by comparing AUC over all the preprocessing methods. This was dominated by the mask-out tiles with 7 of the top 10 using this preprocessing method. The remaining three were made up of two original tiles and one segmented. With the addition of Swin-Tiny/4 MIL results, DTFD mask-out achieves the highest single-model AUC (0.96) and ranks first overall; MIL models with domain-specific features are well represented in the top tier, alongside the ViT and CNN mask-out models. To further analyze the differences in preprocessing, Figure 4 compares the mean AUC and accuracy between each framework on the original, mask-out, and segmentation sets with their standard deviations. An ANOVA *t*-test was performed to check for significant differences between model framework performance and preprocessing. Surprisingly, no statistically significant differences were observed across all preprocessing methods in the ViT models based on the patient AUCs, with all preprocessing methods having high standard deviations. Accuracy was statistically significantly better between the original ViTs and the segmented ViTs. MIL models performed statistically significantly better on the original tiles than the mask-out and segmented (*P* < 0.001) on both metrics. All CNN, ViT, and MIL frameworks performed statistically significantly (*P* < 0.05) better on the original tiles in comparison with the segmented when looking at accuracy (Fig. 4b).

**Figure 3.**
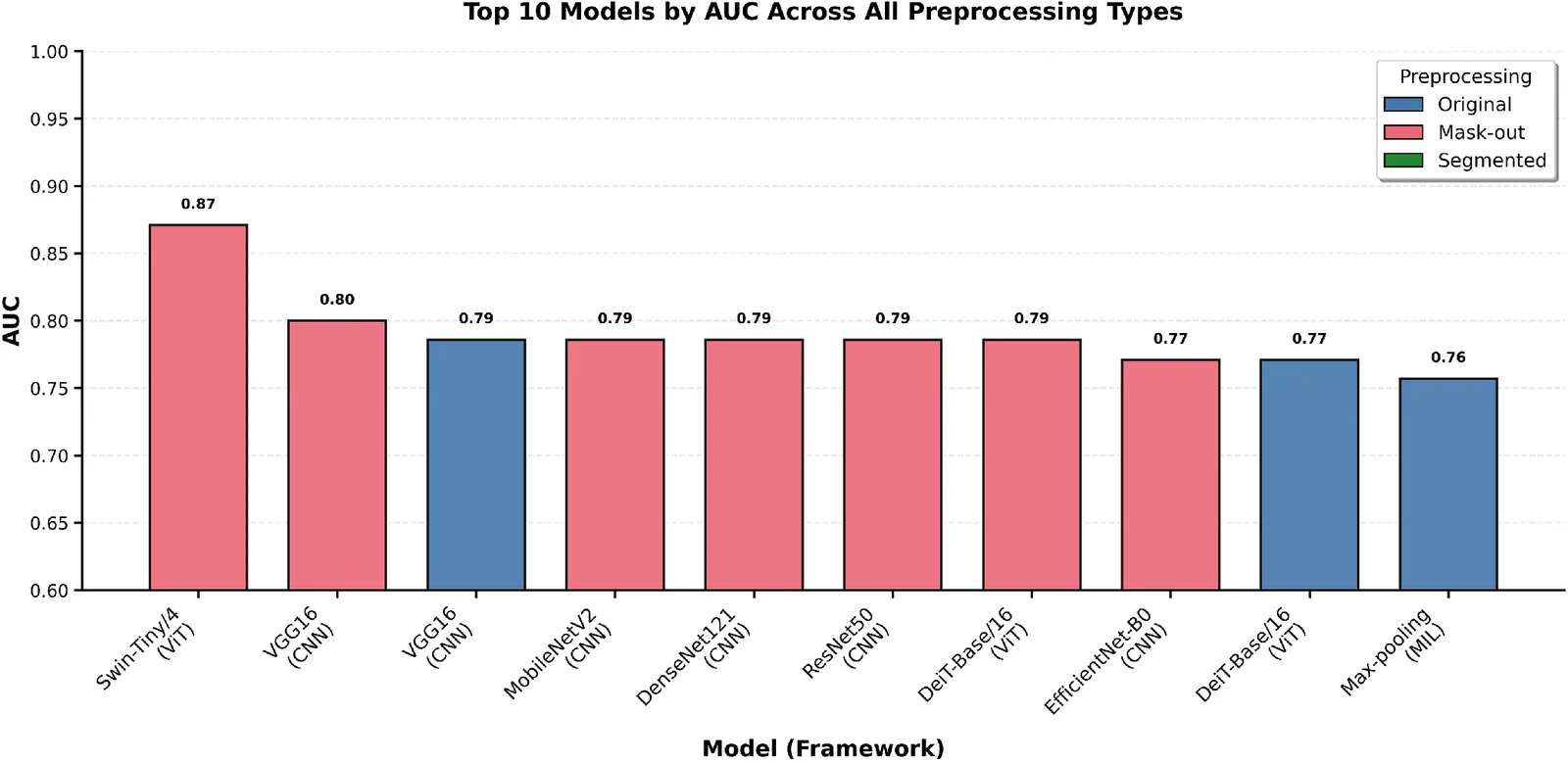
Top 10 best-performing models across all frameworks and preprocessing tests.

**Figure 4.**
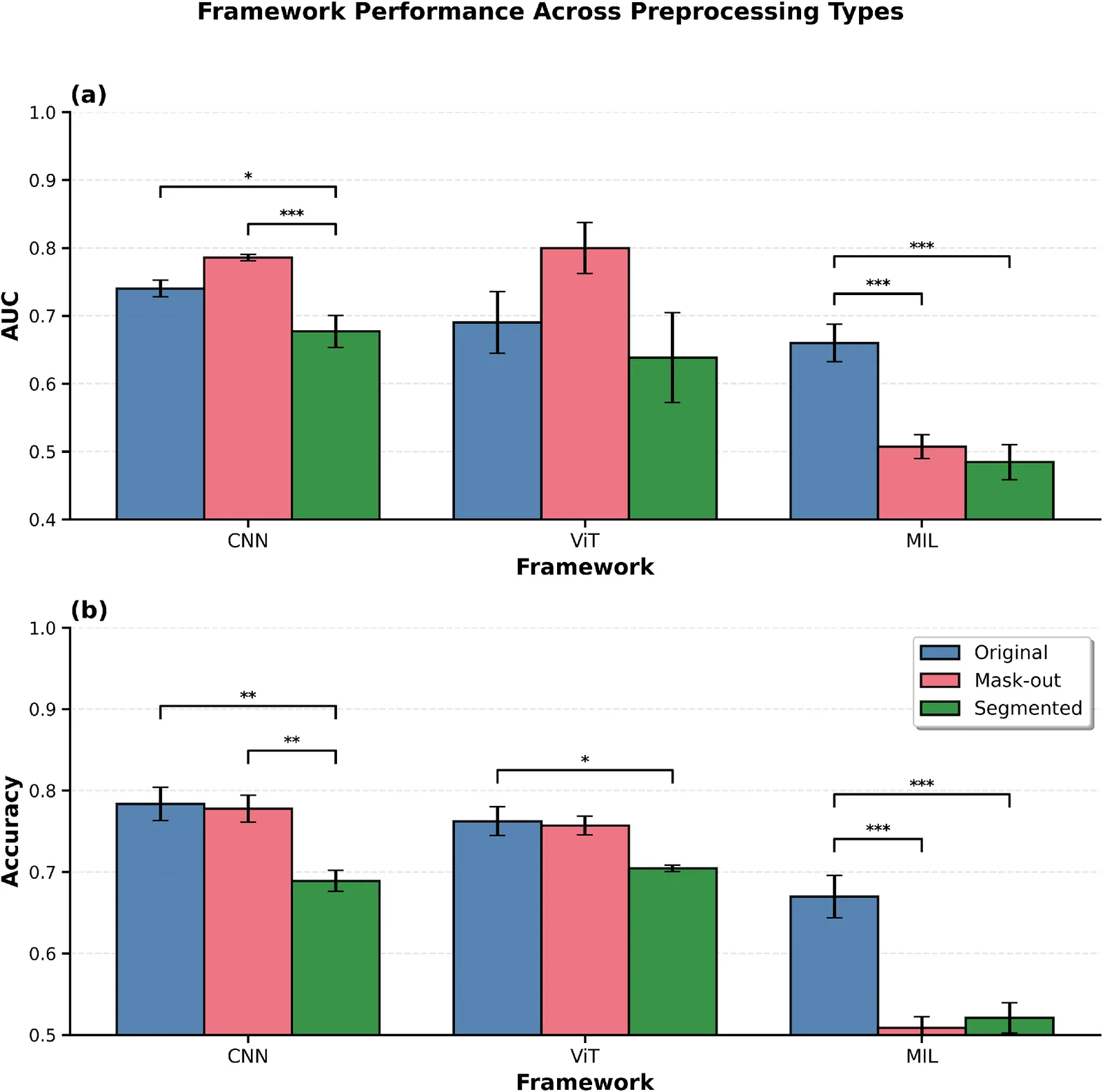
Comparison of different frameworks and preprocessing steps. (**a**) The mean AUC of the per framework. (**b**) The mean accuracy per framework. Error bars represent standard deviation. Statistical significance determined by ANOVA *t*-test: *P *<* 0.05, **P *<* 0.01, ***P *<* 0.001.

### Misclassification Analysis

To understand the failure modes of the best-performing model (Swin-Tiny/4, AUC 0.87), misclassified tiles and patients from the held-out internal test set were examined. Two mCA patients were misclassified as CM, with 59.3% and 76.1% of tiles incorrectly identified. One case of CM was identified incorrectly as an mCA with 65.4% of tiles incorrectly classified. A manual analysis of the misclassified patients revealed that the misclassified CM patient had a sparse sample with highly variable cellularity and very little pigmentation. Of the two misclassified mCA patients, both were lung carcinomas. Both samples were very sparse, with the first having the presence of some artifacts and the second having a large amount of background staining artifact.

## Discussion

This study applies a range of DL architectures to ocular oncology cytology specimens. The U-Net achieved a recall of 0.76 and precision of 0.80, demonstrating that the model successfully detected most tumor cells with a mean localization error of 1.91 µm. DTFD-MIL^20^ with mask-out preprocessing achieved the best patient-level AUC of 0.96 (95% confidence interval, 0.83–1.00) using the fine-tuned Swin-Tiny/4 backbone, with the Swin-Tiny/4 ViT achieving an AUC of 0.87 with the same preprocessing, both demonstrating a strong diagnostic performance for this task. Overall, the AUC performance suggests that the task can be learned across several architectures and frameworks. Full segmentation, which removes all background context, resulted in the worst classification performance across the CNN and ViT frameworks, a finding that was statistically significant (*P* *<* 0.05). The trend toward a better performance with mask-out tiles compared with original tiles did not attain statistical significance at this sample size and should therefore be interpreted cautiously. The consistent finding across models is that stripping all noncellular context is harmful, while retaining a local margin around segmented cells is at least as good as, and in several models numerically better than, using the unprocessed tile. U-Net segmentation and then expanded masks also reduces the computational burden by removing unnecessary features, decreasing the tile file size, and improving the training and inference times. The statistically supported finding regarding preprocessing is that full segmentation is detrimental to classification. The consistent trend toward better performance with mask-out tiles is plausible and likely reflects the preservation of the local cellular context, but it did not attain statistical significance and should be regarded as a hypothesis rather than a confirmed conclusion at this sample size. The likely mechanism is that relevant spatial context is lost during full segmentation: background features, including cell spacing, density patterns, and the presence of nontumor elements such as macrophages, lymphocytes, and red blood cells, carry diagnostically relevant information that is removed during segmentation. For example, the spatial distribution of cellular elements across the slide may itself contribute to classification, as has been recognized in other cytological domains. Retaining a local margin around segmented cells, as in the mask-out approach, preserves the pericellular context that appears to be useful for classification. MGG staining produces color gradients that extend to the cytoplasmic boundary, where intensity could potentially differ between CM and mCA cases. Forcing the models to focus solely on the isolated morphology of the tumor cells eliminates features that would otherwise be used in by the model for classification. The U-Net should be used as a preprocessing step to reduce the number of tiles, keeping the mask-out tiles that matter. Beyond the effect of preprocessing, the choice of model architecture also had a substantial impact on performance. The misclassification analysis further supports this finding, with the model struggling most on sparse, low-cellularity samples and cases with a mixed reaction to the pigment. Furthermore, the centroid-based circular masks used in this study primarily capture nuclear regions with limited cytoplasmic morphology, meaning that diagnostic features such as melanin granules and cytoplasmic mucin may be partially excluded. Instance segmentation approaches such as Cellpose^22^ or HoVer-Net,^23^ which delineate complete cell boundaries, could better preserve these features in future work. Interframework comparison shows that MILs with domain-specific features and ViTs achieved the strongest overall performance, with CNN architectures performing moderately. MIL aggregators were trained using precomputed features from the fine-tuned Swin-Tiny/4 backbone, providing task-specific representations for the aggregation step. DTFD achieved the highest single-model AUC in the entire comparison at 0.96 (95% confidence interval, 0.83–1.00) on mask-out tiles, and all aggregators except Max achieved an AUC of 0.86 or greater on mask-out tiles. On original tiles, Max and Attention aggregation tied for best (AUC, 0.81). On segmented tiles, most MIL aggregators showed near-zero sensitivity with perfect specificity, consistent with model collapse when all local pericellular context is removed. However, all models were trained under otherwise identical conditions with the same data, epochs, and optimizer. Performance differences for the CNN and ViT therefore likely reflect variations in how effectively each pretrained model transferred to this specific task rather than fundamental architectural advantages for sparse cytological data. VGG16 (AUC, 0.80) and Swin-Tiny/4 (AUC, 0.87) demonstrated superior CNN/ViT performance, likely because lighter architectures require fewer parameters for effective fine-tuning on limited cytospin data, whereas deeper models demand larger datasets and more extensive hyperparameter optimization to avoid overfitting. Given the small dataset size (87 patients), no conclusions about which framework is best suited to all cytological specimens can be drawn, as other studies have reported different rankings across architectures on similarly sparse preparations.^24^ It is difficult to compare this study with existing studies due to the scarcity of classification and segmentation, not only in ocular oncology, but also using the MGG stain. The most substantial work in this area was by Matek et al.,^25^ applying DL models to MGG-stained bone marrow cytological specimens. They developed a highly accurate differentiation tool with bone marrow being the most tested cytological samples. In uveal melanoma, Liu et al.^26^ developed a DL system to predict the gene expression profile class of the tumor cells directly from digital cytopathology WSIs of fine-needle aspiration biopsy specimens, demonstrating the feasibility of AI-based prognostication from cytological material. However, their approach focused on slide-level gene expression profile predictions rather than individual cell segmentation and classifications. Similar to classification, the application of segmentation models to MGG-stained cytospins has never been performed in ocular oncology. This factor highlights the novel nature of this study, creating a full pipeline of tumor cell segmentation and classification together. The primary implication of this study is the potential development of a decision support tool, to aid specialist ocular pathologists in their diagnosis, particularly for ambiguous cases where the class activation maps could highlight regions of interest for further review. This tool would serve as a complementary aid to the pathologist, with the primary goal of improving diagnostic accuracy, but would not replace specialist expertise. In centers without dedicated ocular pathology expertise, such a tool could support triage by flagging cases that require referral to a specialist center, although expert oversight remains essential for the final diagnosis.

The held-out test set comprised only 17 patients, which is a direct consequence of the rarity of intraocular biopsy material. Bootstrapped 95% confidence intervals (2000 resamples, patient level) for all reported test set metrics are wide; for example, the best-performing model overall (DTFD-MIL mask-out; AUC, 0.96) has a 95% confidence interval spanning 0.83 to 1.00, and the best ViT (Swin-Tiny/4 mask-out; AUC, 0.87) has a 95% confidence interval spanning 0.64 to 1.00, confirming that point estimates from this sample size carry substantial uncertainty and that rankings between models should not be overinterpreted. The fold selection strategy (reporting the model from the fold with the highest validation AUC) means that variability across folds is not captured in the reported metrics, and the selected fold may be somewhat optimistically biased compared with a fold-averaged estimate. The held-out internal test set remains unbiased, as it was not involved in fold selection.

Due to the rarity of intraocular biopsies, the small patient cohort of 87 patients from a single center limits the generalizability of the models. Additionally, only a single scanner and protocol was used, where other centers may prepare the stain in a different way and other scanners are known to produce different color intensities. At the patient level, there is a slight class imbalance of 50 CMs vs 37 mCAs, which could impact the performance and why the AUC was chosen as the primary performance metric. Manual annotation was also performed by a single trained person; therefore, there was no assessment of interobserver variability.

Several methodological limitations should also be noted. The U-Net–based filtering step, which retains only tiles with 5 or more detected tumor cells, is a strong selection mechanism: of 742,023 nonbackground tiles extracted after white space removal, 231,677 (31.2%) were retained, meaning that 68.8% of the tiles were excluded. This enriches the dataset for tumor-dense, morphologically informative regions and may inflate performance estimates relative to real-world conditions where low-cellularity or degraded specimens are common. Paucicellular slides, precisely the diagnostically challenging cases, are likely under-represented, which is consistent with the misclassification analysis, where sparse samples contributed disproportionately to errors. The five or more cell threshold could be treated as a tunable parameter in future work. The U-Net was trained without an explicit nontumor class, meaning that it may detect morphologically similar nontumor cells, and future iterations could incorporate multiclass annotations to address this. Additionally, using the U-Net to filter tiles introduces a selection bias, as the classification dataset preferentially contains tiles with separable cells resembling the U-Net training data, excluding tiles with heavily overlapping clusters or very few cells. Although this filtering was necessary to remove the majority of the uninformative background tiles and reduce the computational burden, the classifiers were therefore trained and evaluated on a filtered subset rather than the full range of tiles within each WSI. External validation on an independent cohort is not currently feasible: trans-scleral fine-needle aspiration biopsy of intraocular tumors is a highly specialized procedure, and MGG-stained cytospin WSI datasets of this type do not exist at other centers to our knowledge. The Liverpool Ocular Oncology Center is one of the largest dedicated units globally, and this 87-patient cohort accumulated over more than a decade. Multicenter collaboration is being pursued and external validation will be the focus of a follow-up study.

Multimodal AI could further enhance performance by incorporating standard ophthalmic imaging modalities such as fundoscopy, optical coherence tomography, or ultrasound examination alongside diagnostic biopsies. This work could also serve as a foundation for genetic risk stratification from biopsy material, enabling a fully integrated diagnostic and prognostic pipeline. In summary, this framework establishes a foundation for AI-assisted cytopathology in ocular oncology that could improve diagnostic accuracy and support specialist pathologists in their clinical workflow. It is a step forward in the application of AI in both ocular oncology and cytopathology, which could lead to an increased number of applications in the future.
